# Supplementary material for: The concerted action of SEPT9 and EPLIN modulates the adhesion and migration of human fibroblasts
Source: Life Sci Alliance. 2024 May 7;7(7):e202201686. doi: 10.26508/lsa.202201686 (PMC11077590; doi:10.26508/lsa.202201686)

Raw Fig S1_A

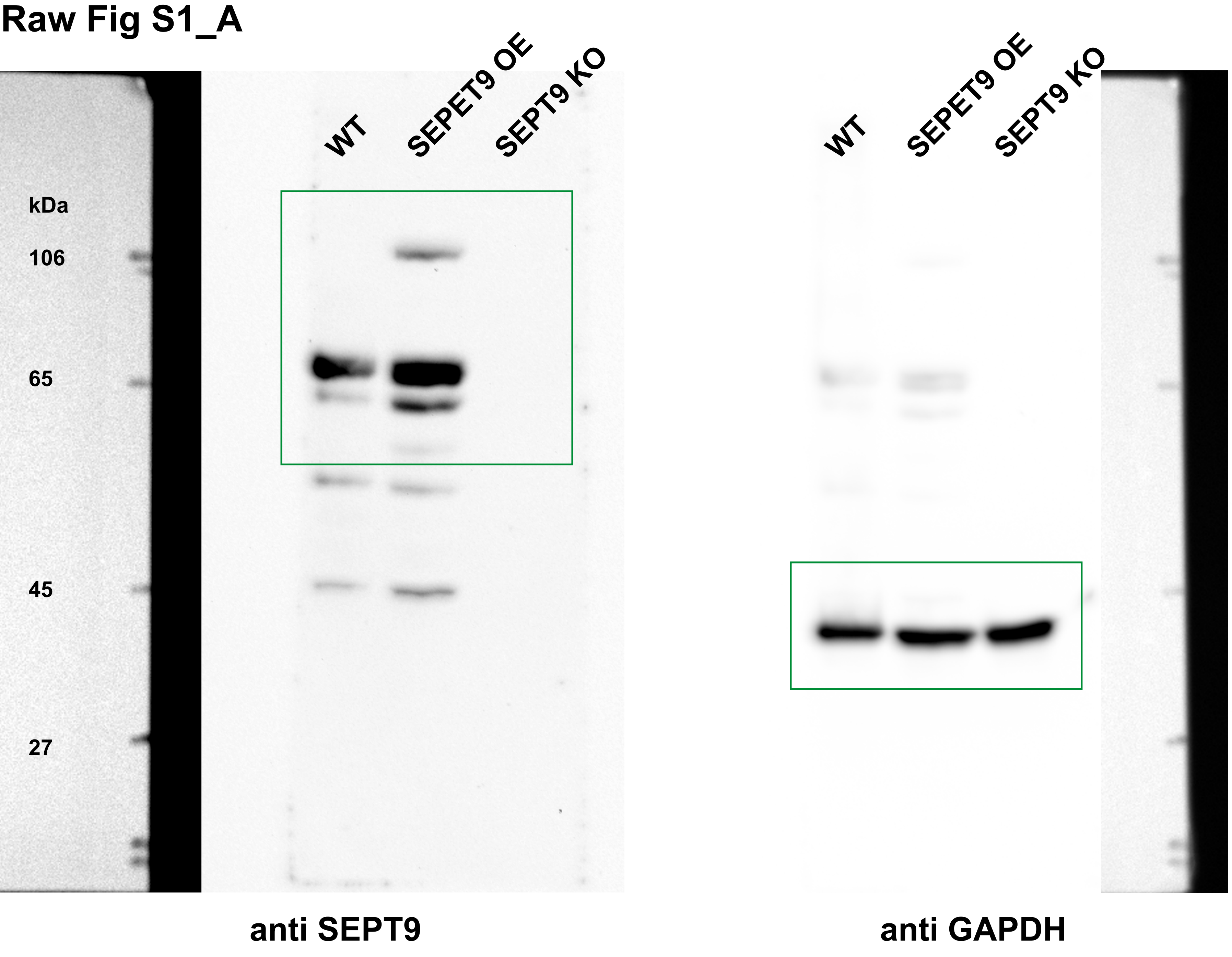

Raw Fig S1_B

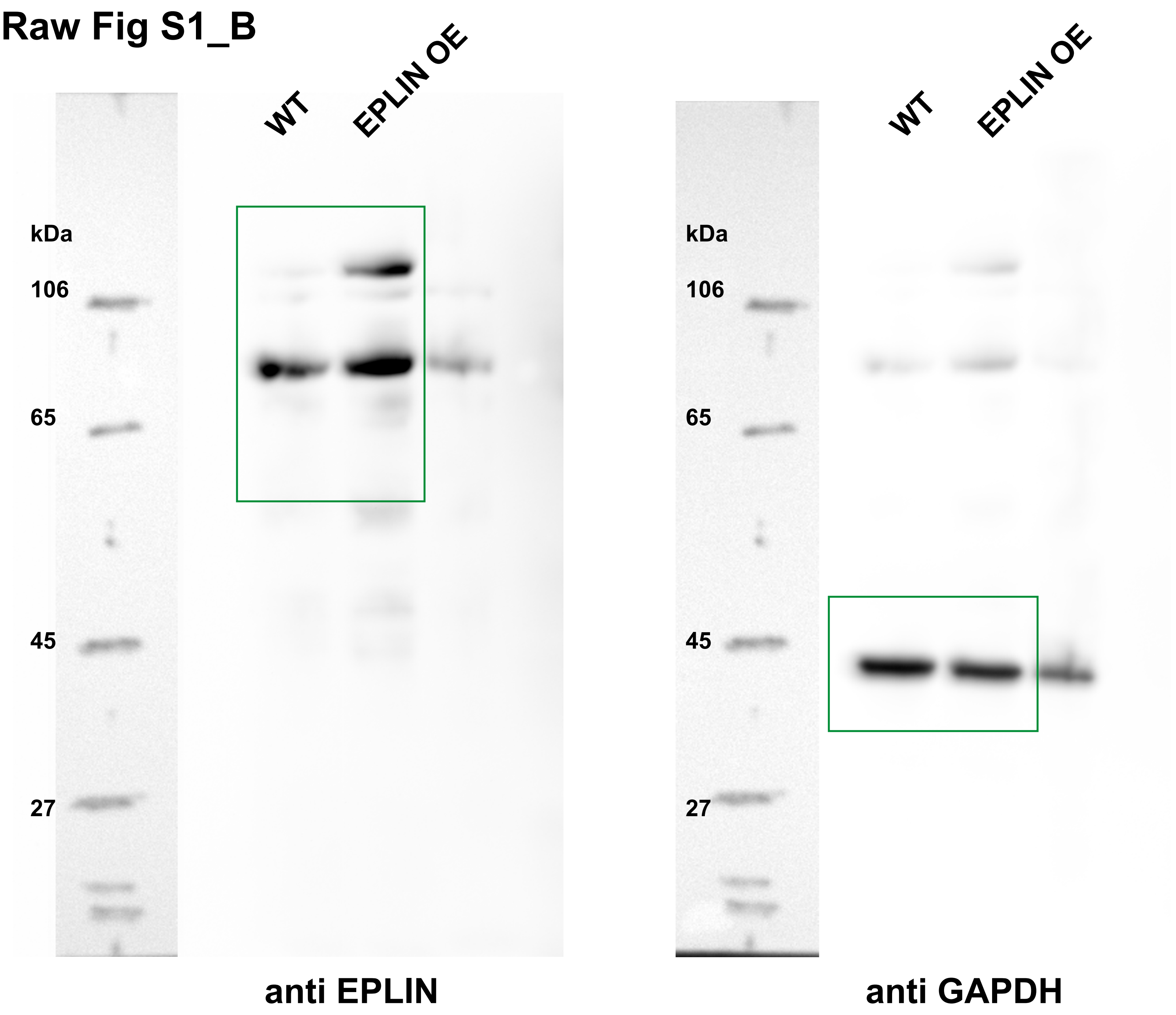

Raw Fig S1_C

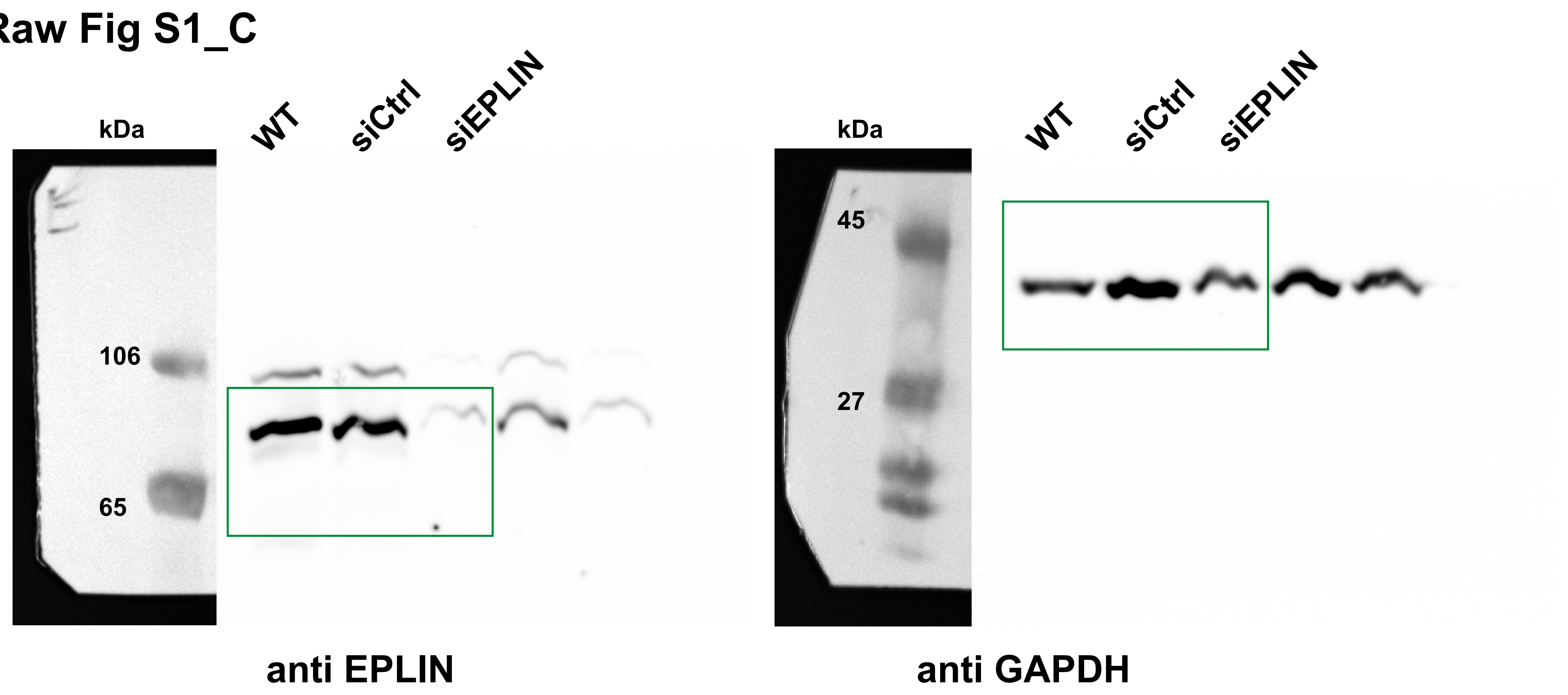

Raw Fig S1_D

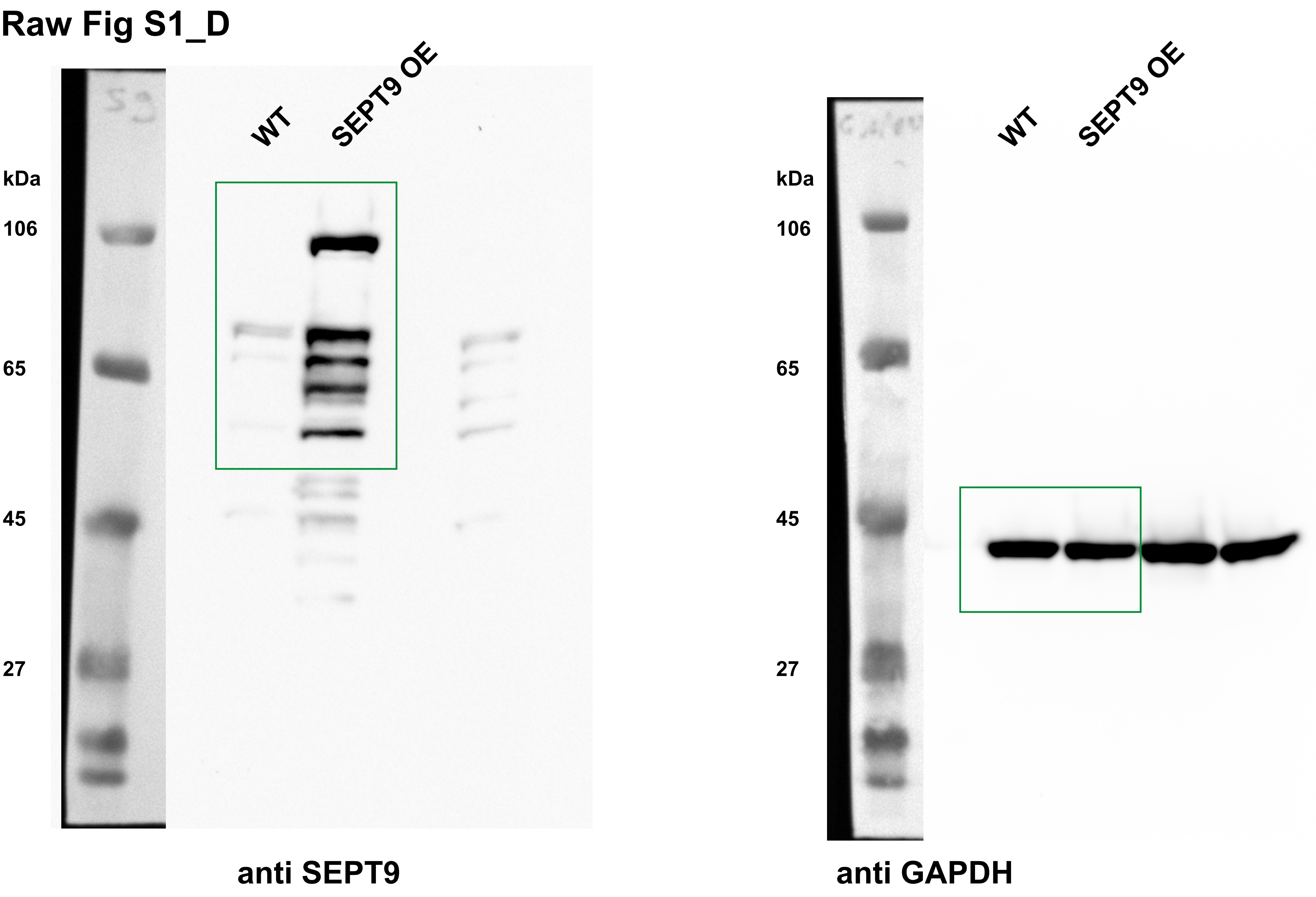

Raw Fig S1_E

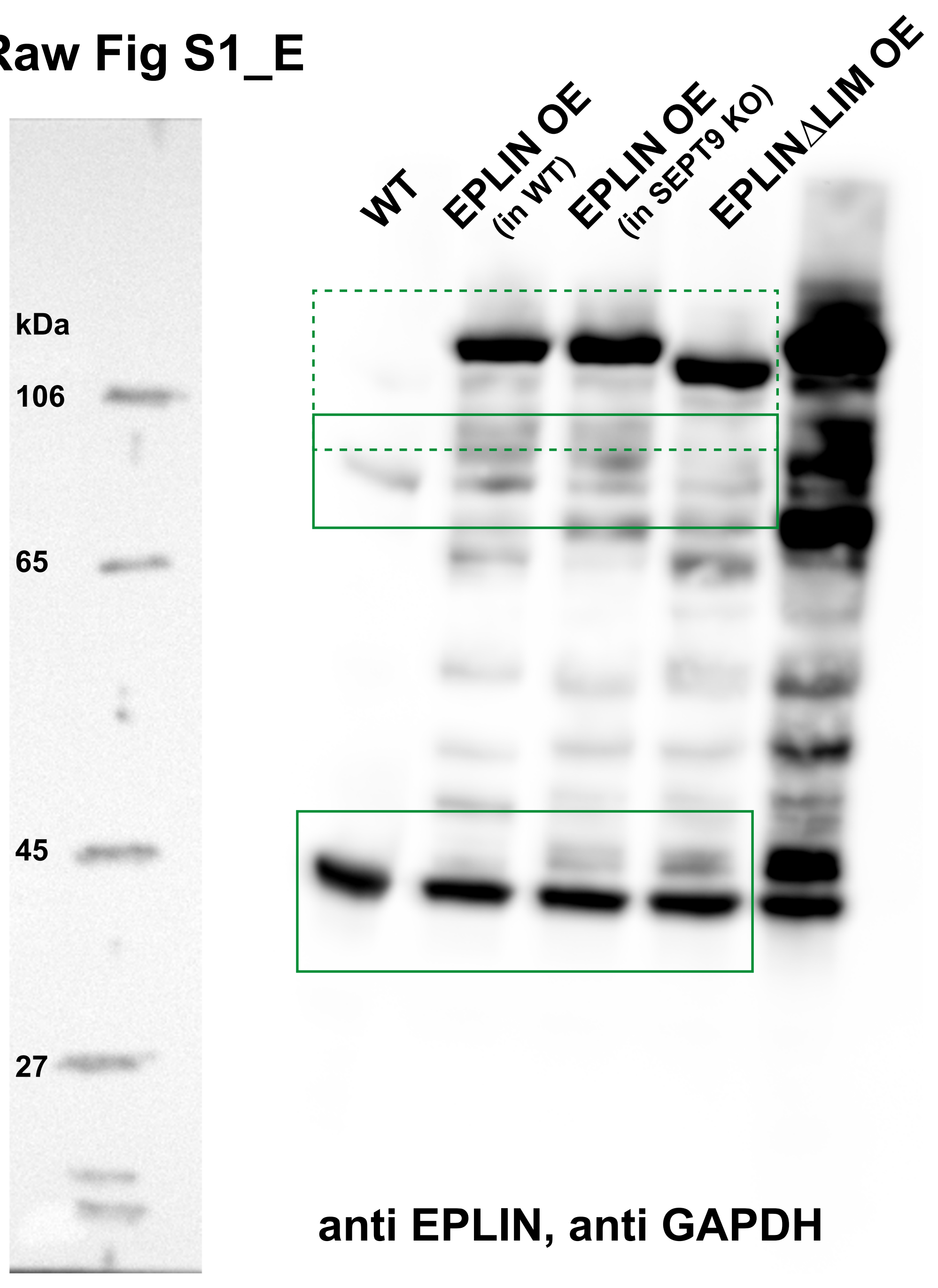

Supplement: Supplementary file 1 [file LSA-2022-01686_SdataF1_FS2_FS3.zip › Raw blots and source data Kopie/SourceDataFigS2.pdf]
