# Supplementary material for: The concerted action of SEPT9 and EPLIN modulates the adhesion and migration of human fibroblasts
Source: Life Sci Alliance. 2024 May 7;7(7):e202201686. doi: 10.26508/lsa.202201686 (PMC11077590; doi:10.26508/lsa.202201686)
Supplement: Supplementary file 1 [file LSA-2022-01686_SdataF1_FS2_FS3.zip › Raw blots and source data Kopie/SourceDataFigS3.pdf]

Raw Fig S1 B

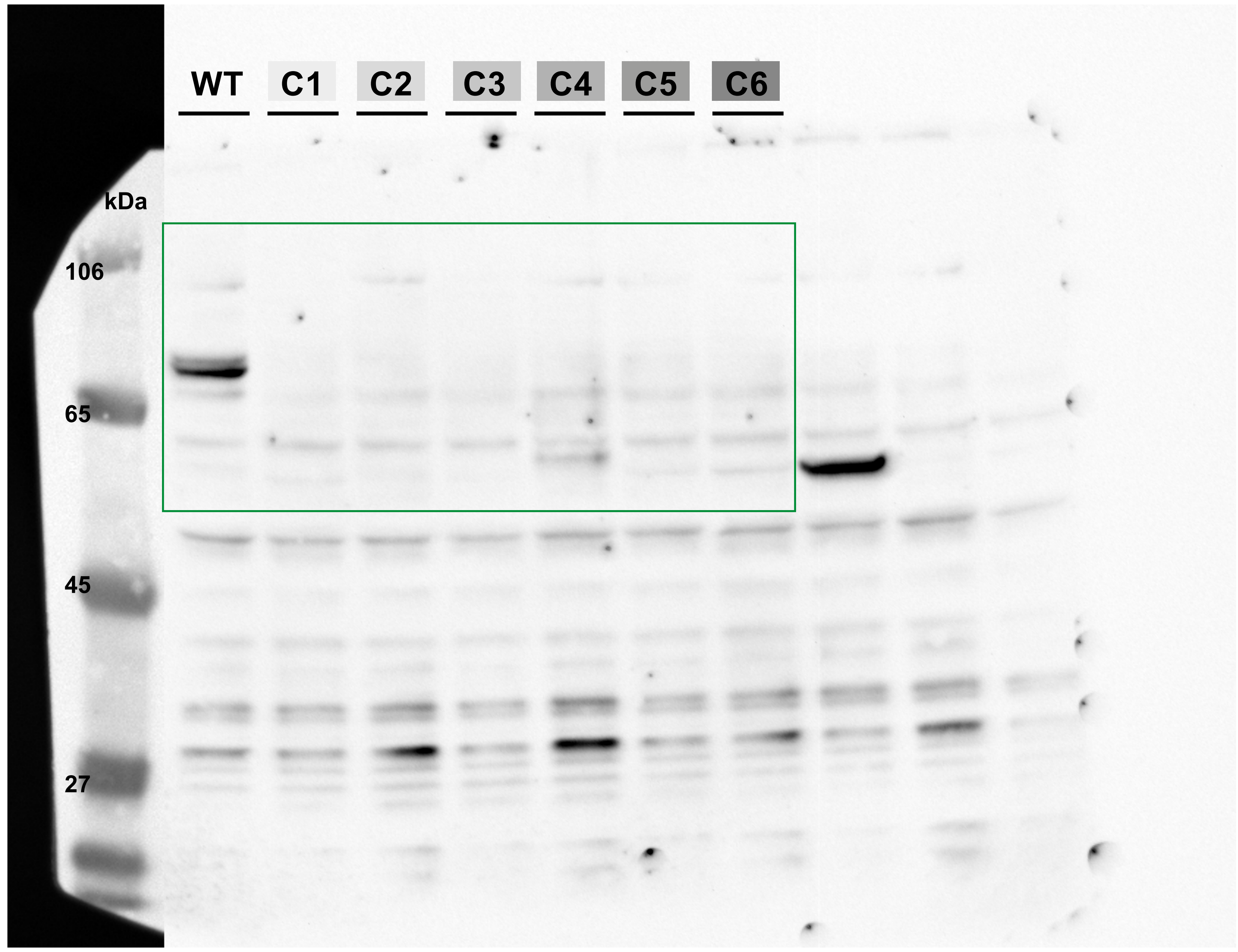

anti SEPT9 (long isoforms)

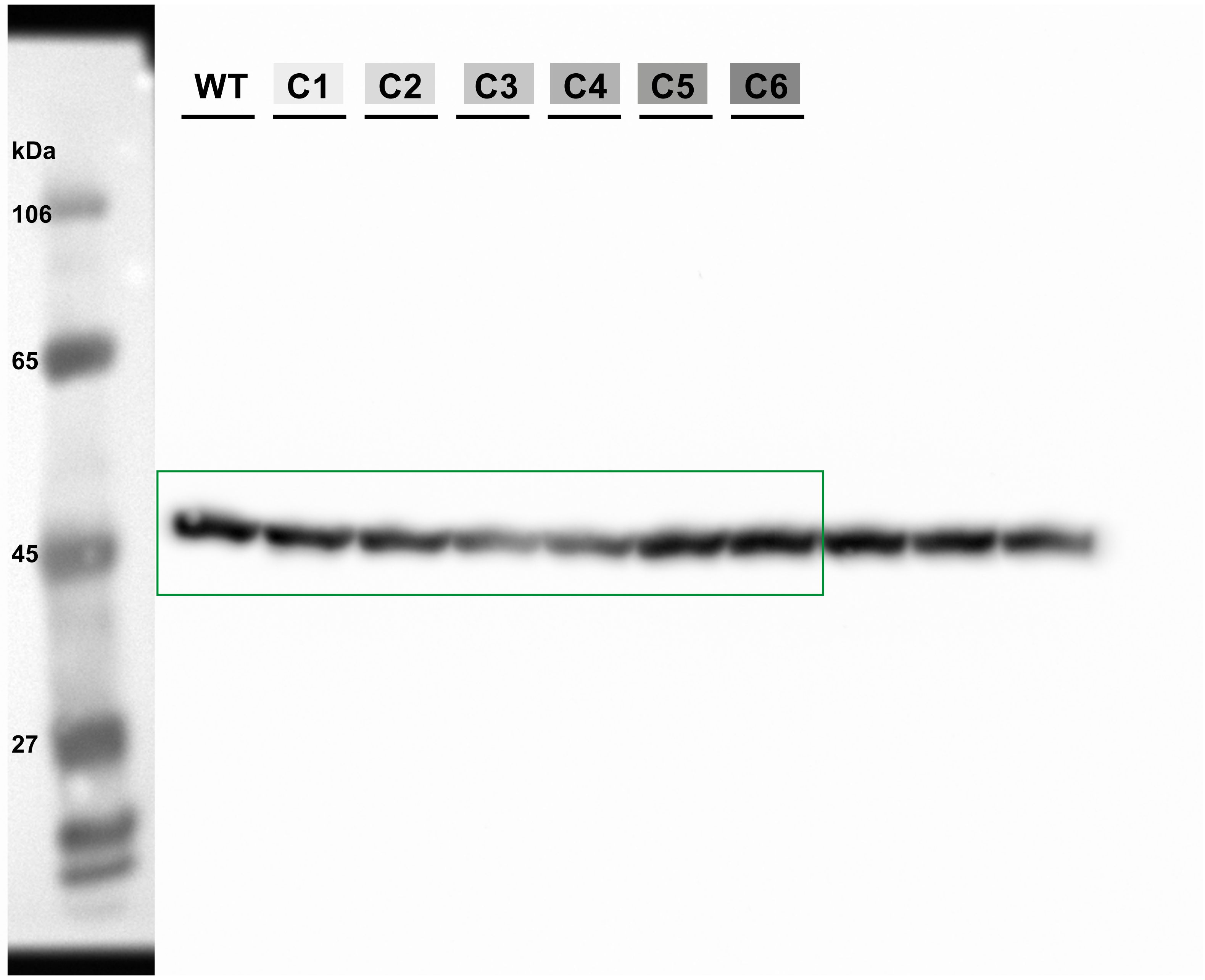

anti Actin

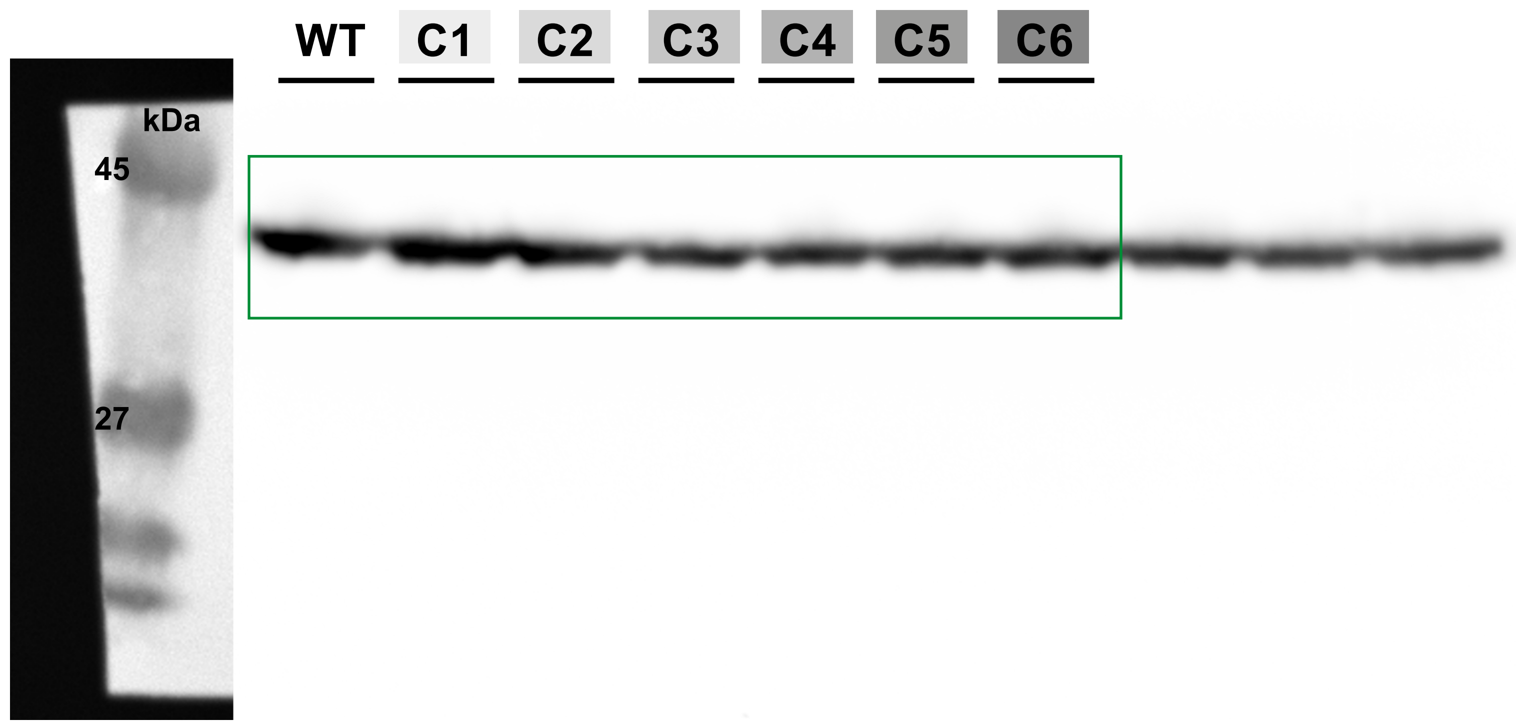

anti GAPDH

Raw Fig S1 E

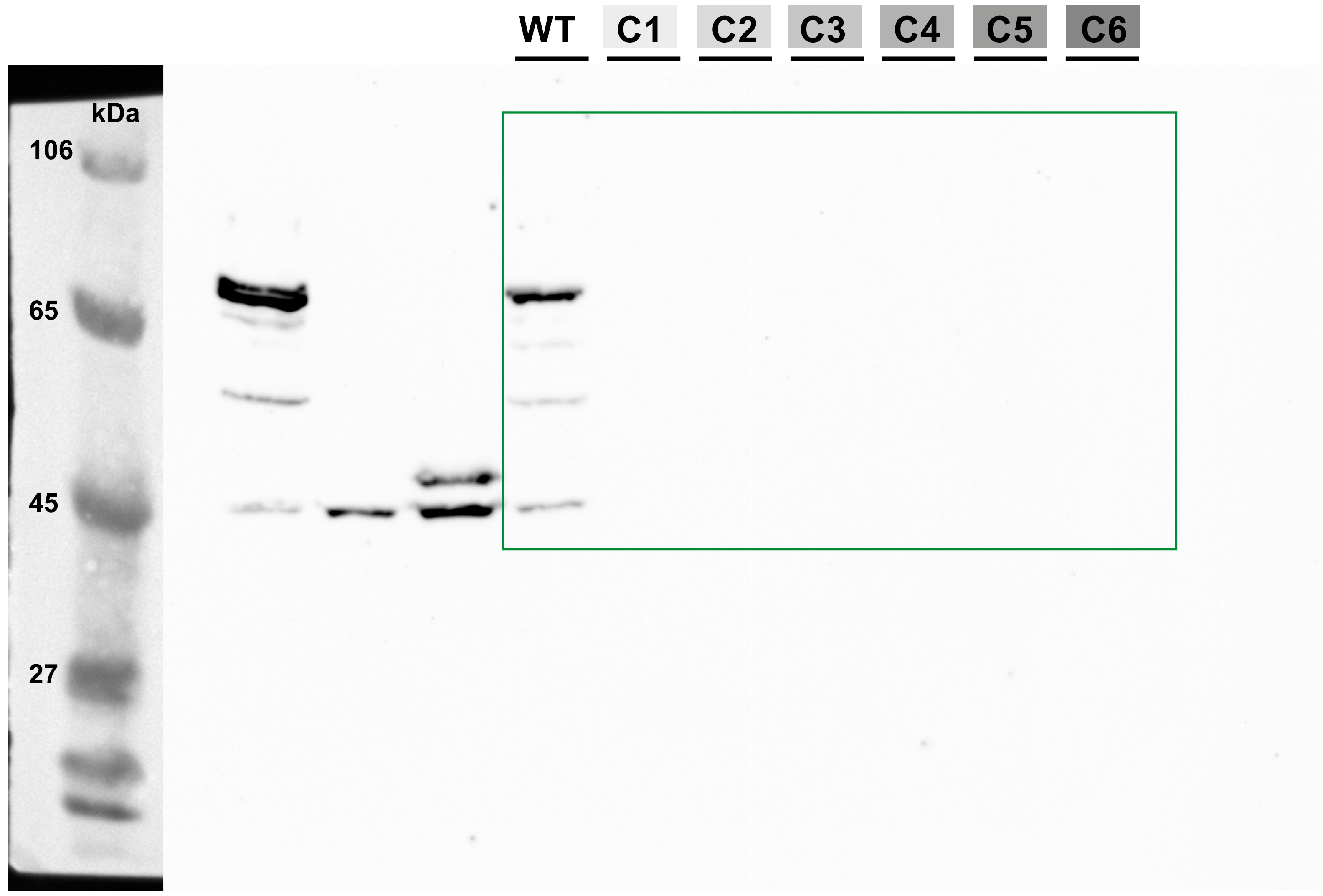

anti SEPT9 (G-domain)

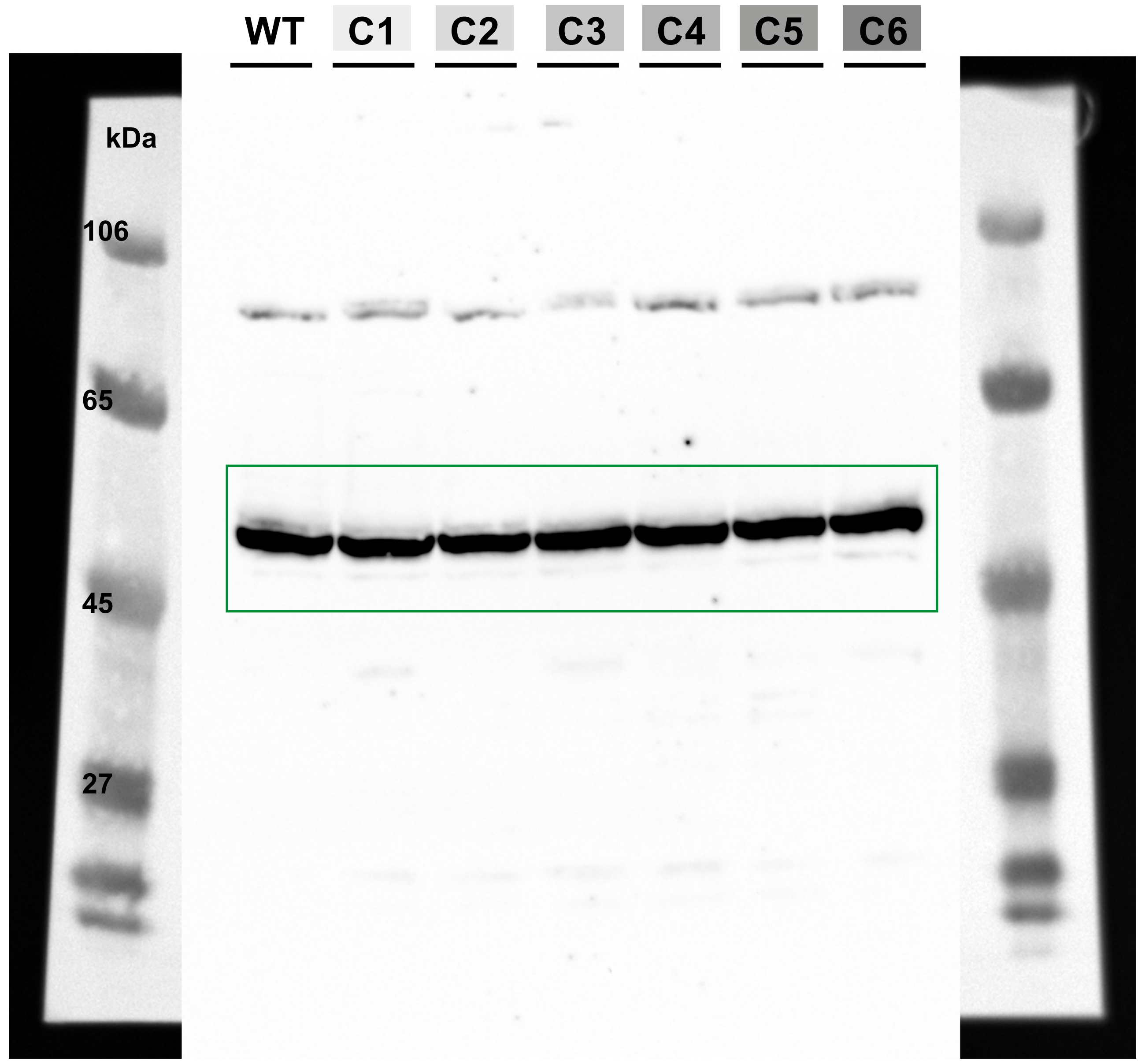

anti SEPT7

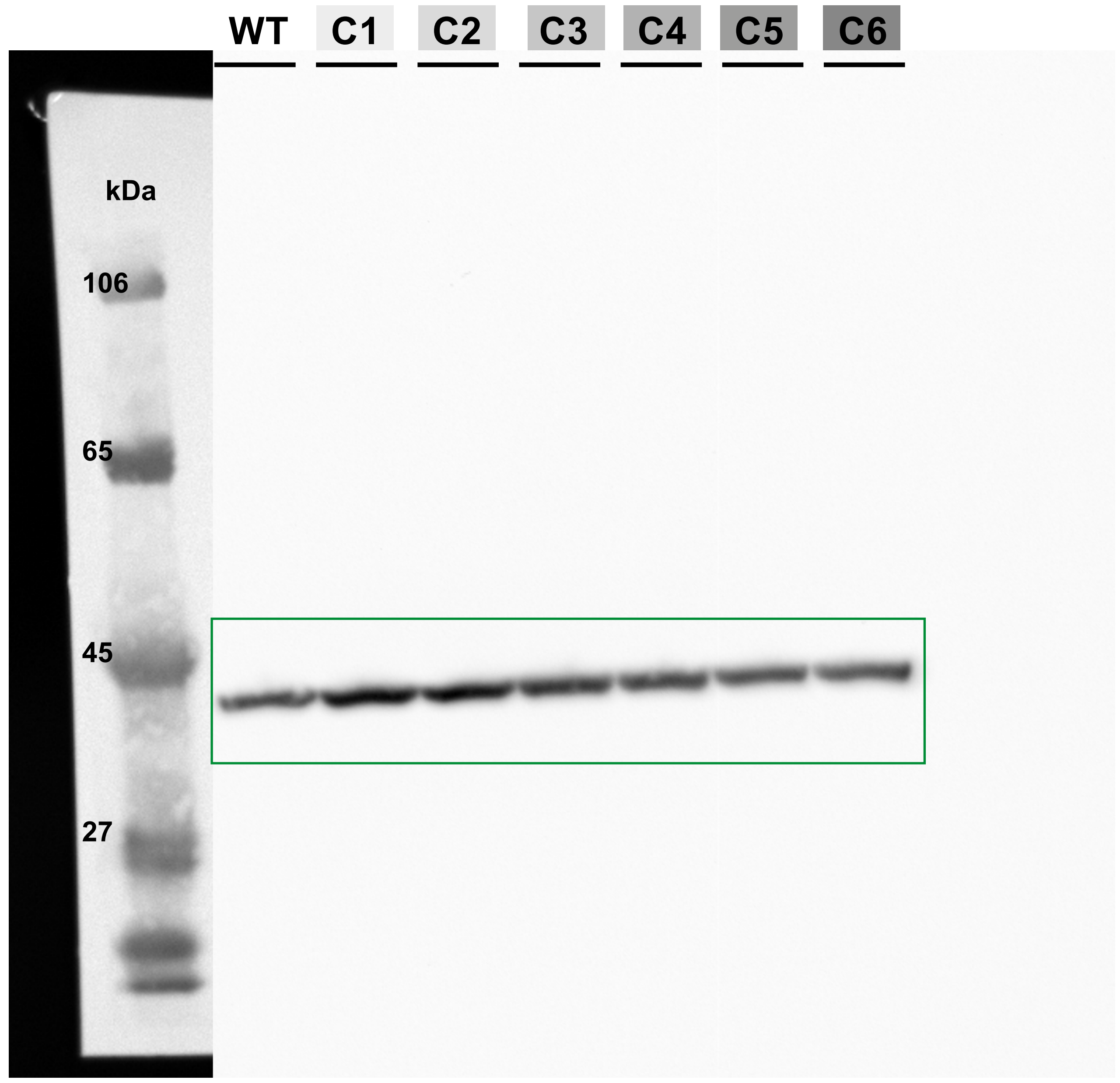

anti GAPDH
