# Supplementary figures and images for: The concerted action of SEPT9 and EPLIN modulates the adhesion and migration of human fibroblasts
Source: Life Sci Alliance. 2024 May 7;7(7):e202201686. doi: 10.26508/lsa.202201686 (PMC11077590; doi:10.26508/lsa.202201686)

Raw Fig 1 C

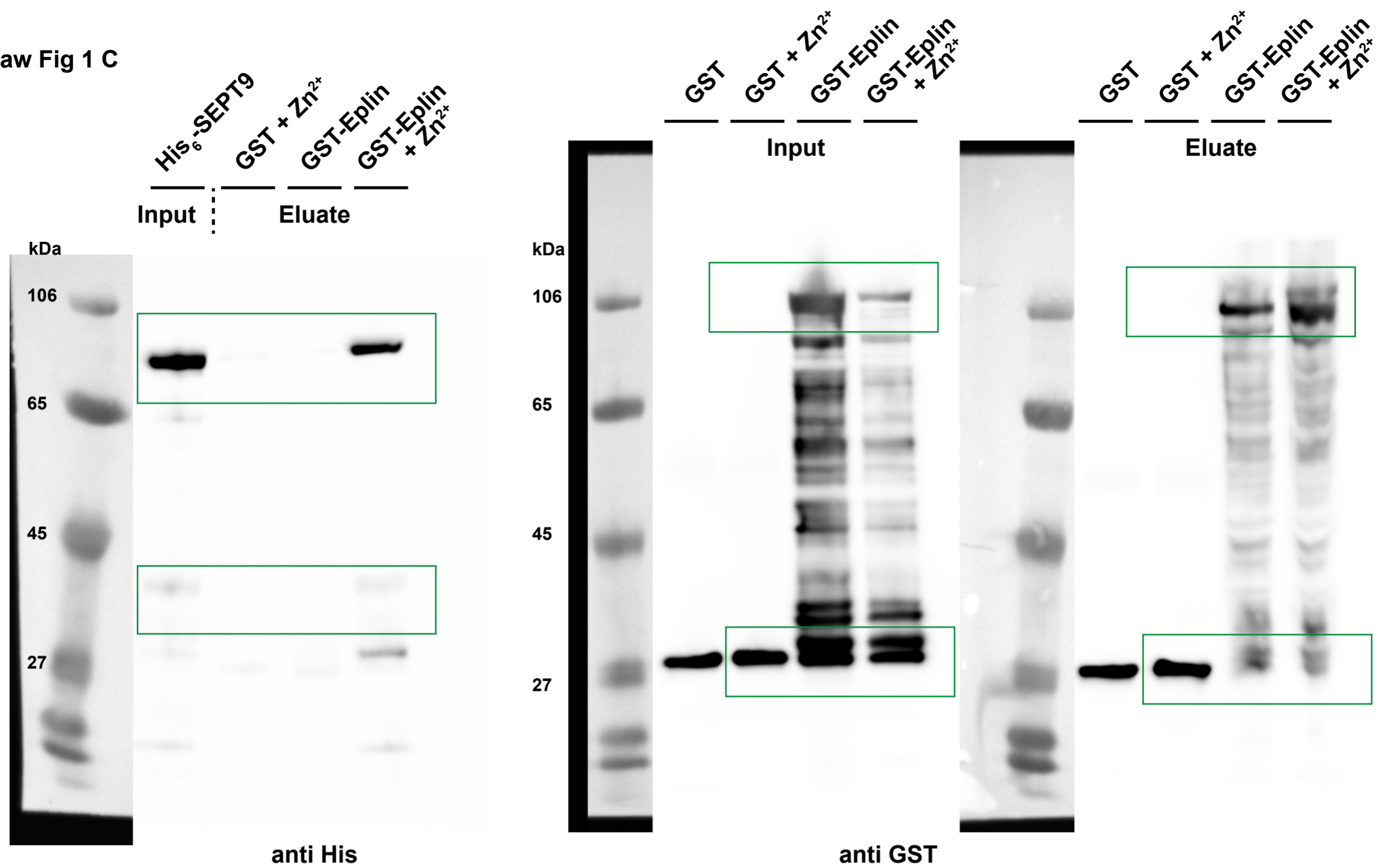

Raw Fig 1 D

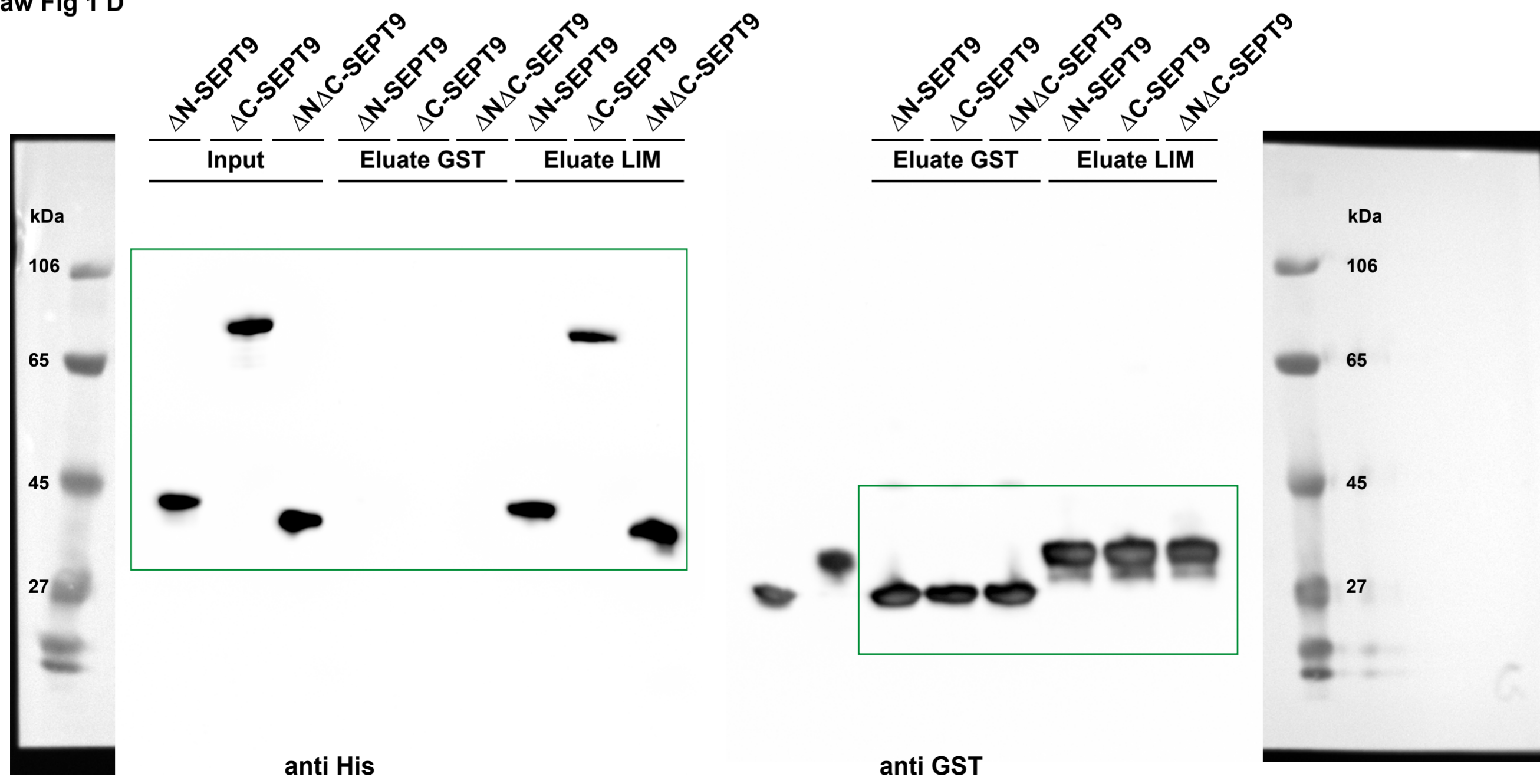

Supplement: Supplementary file 1 [file LSA-2022-01686_SdataF1_FS2_FS3.zip › Raw blots and source data Kopie/SourceDataFig1.pdf]

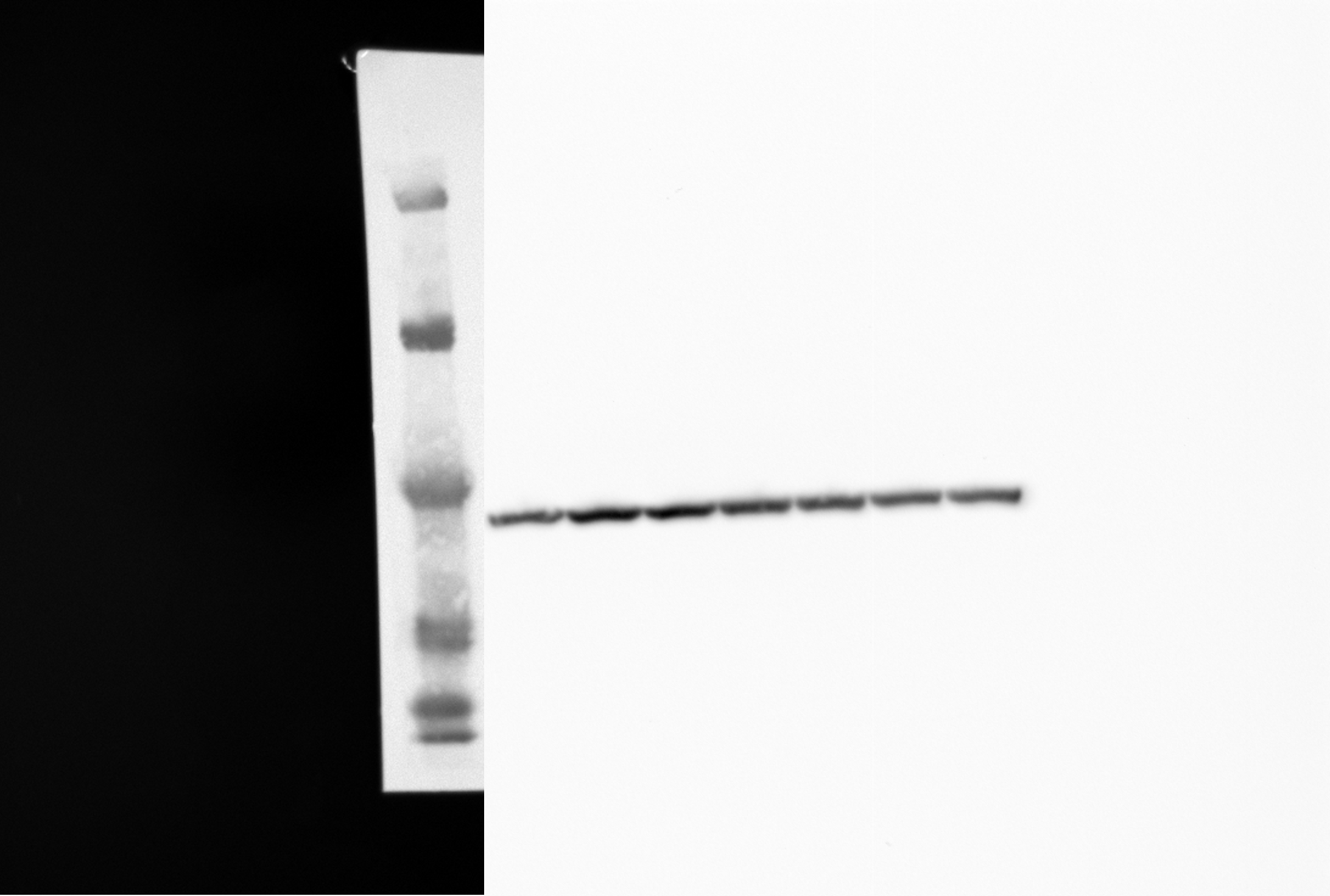

Supplement: Supplementary file 1 [file LSA-2022-01686_SdataF1_FS2_FS3.zip › Raw blots and source data Kopie/Fig S3/S2_E_bottom_Blot 2 GAPDH Banden mit Marker FINAL.png]

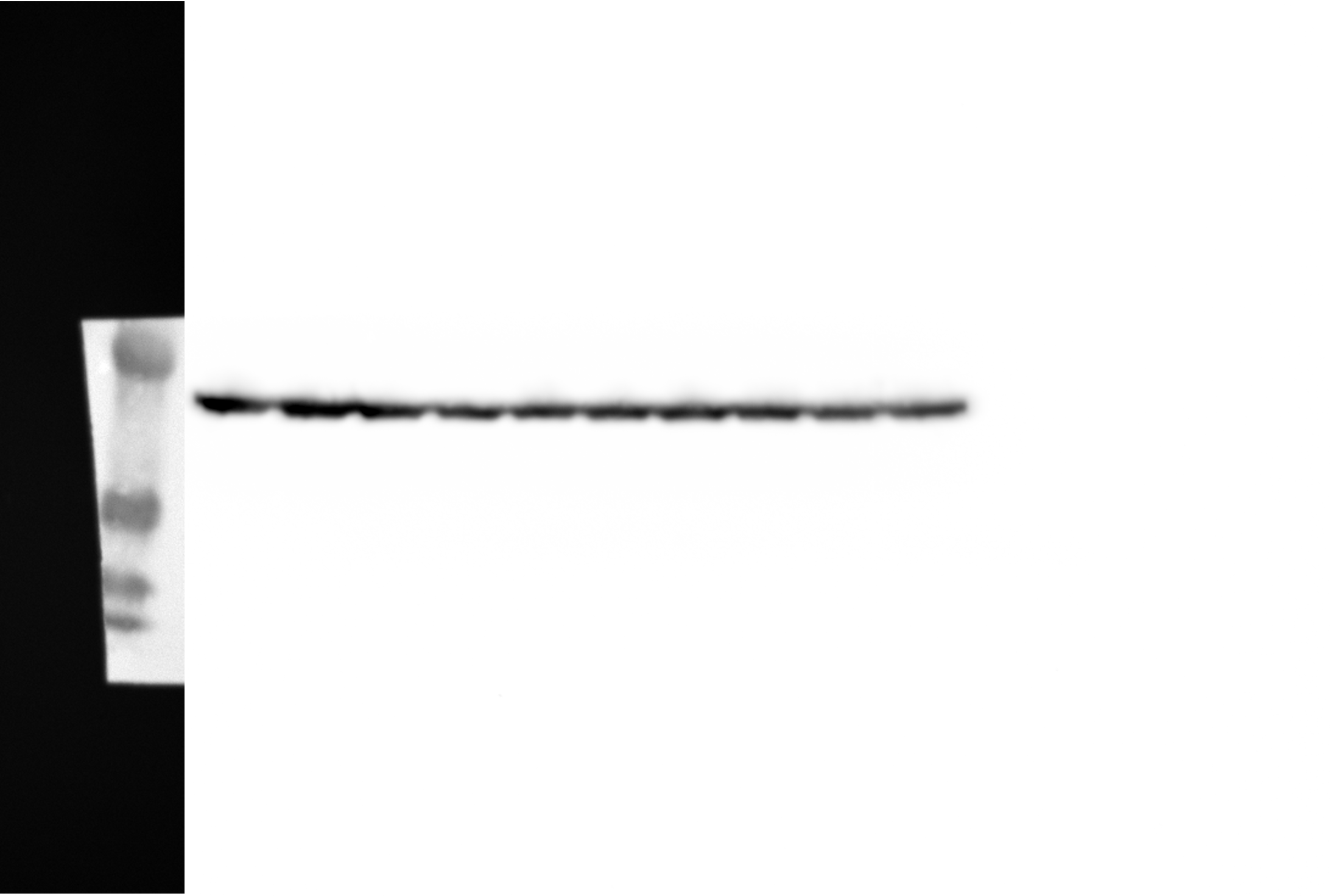

Supplement: Supplementary file 1 [file LSA-2022-01686_SdataF1_FS2_FS3.zip › Raw blots and source data Kopie/Fig S3/S2_B_bottom_Blot 2 medium Banden FINAL.png]

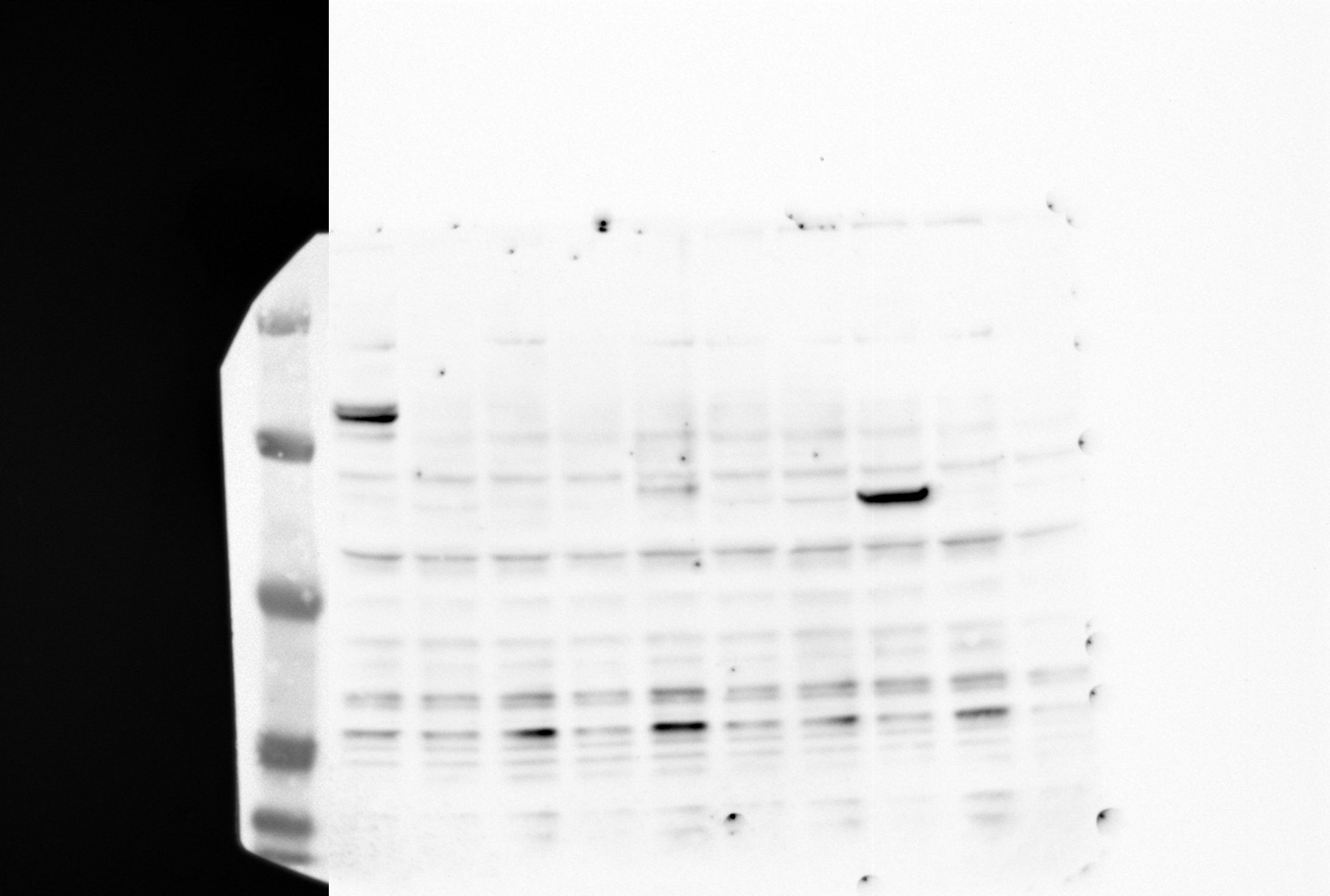

Supplement: Supplementary file 1 [file LSA-2022-01686_SdataF1_FS2_FS3.zip › Raw blots and source data Kopie/Fig S3/S2_B_top_Blot 1 Banden medium FINAL.png]

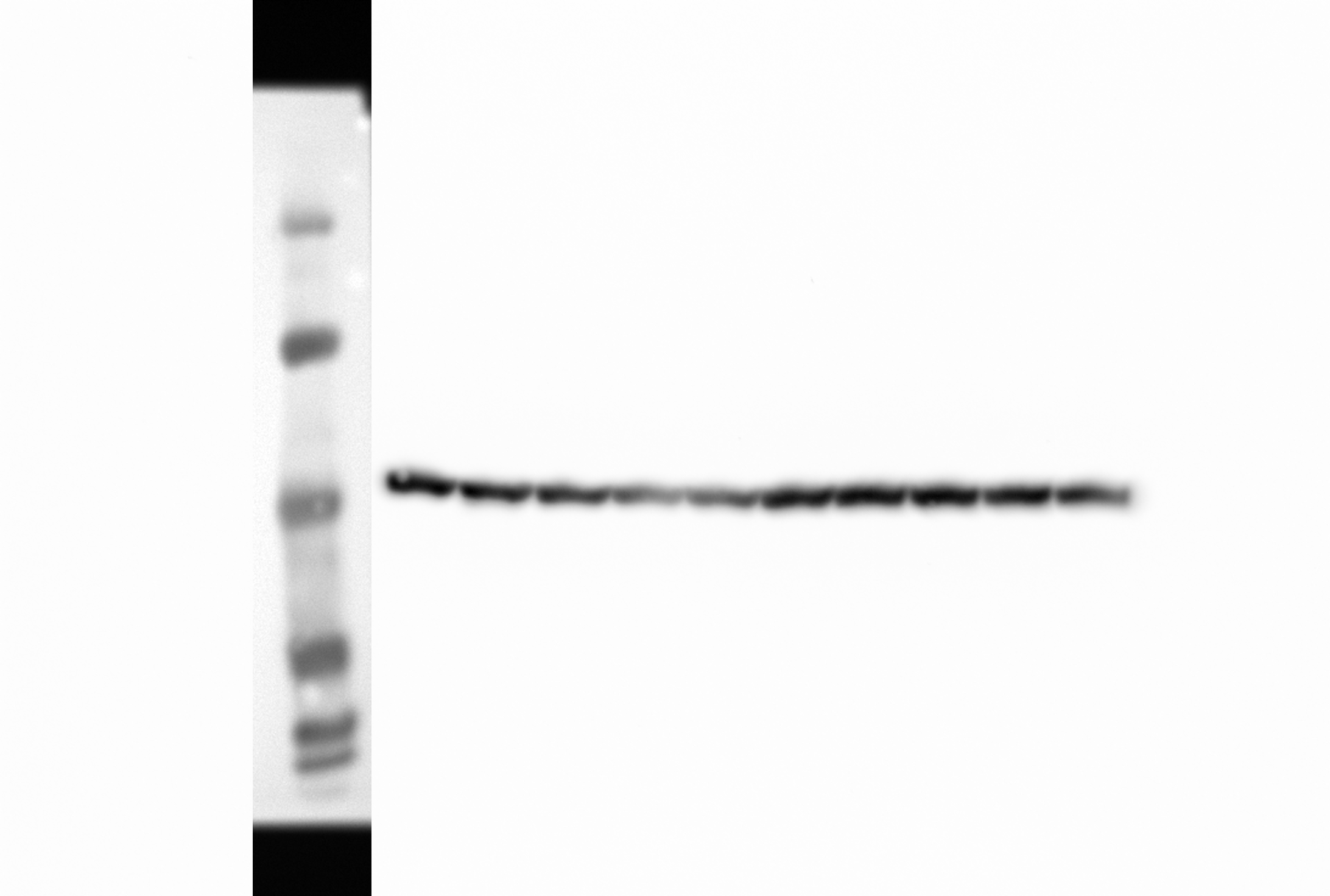

Supplement: Supplementary file 1 [file LSA-2022-01686_SdataF1_FS2_FS3.zip › Raw blots and source data Kopie/Fig S3/S2_B_mid_Actin Banden low FINAL.png]

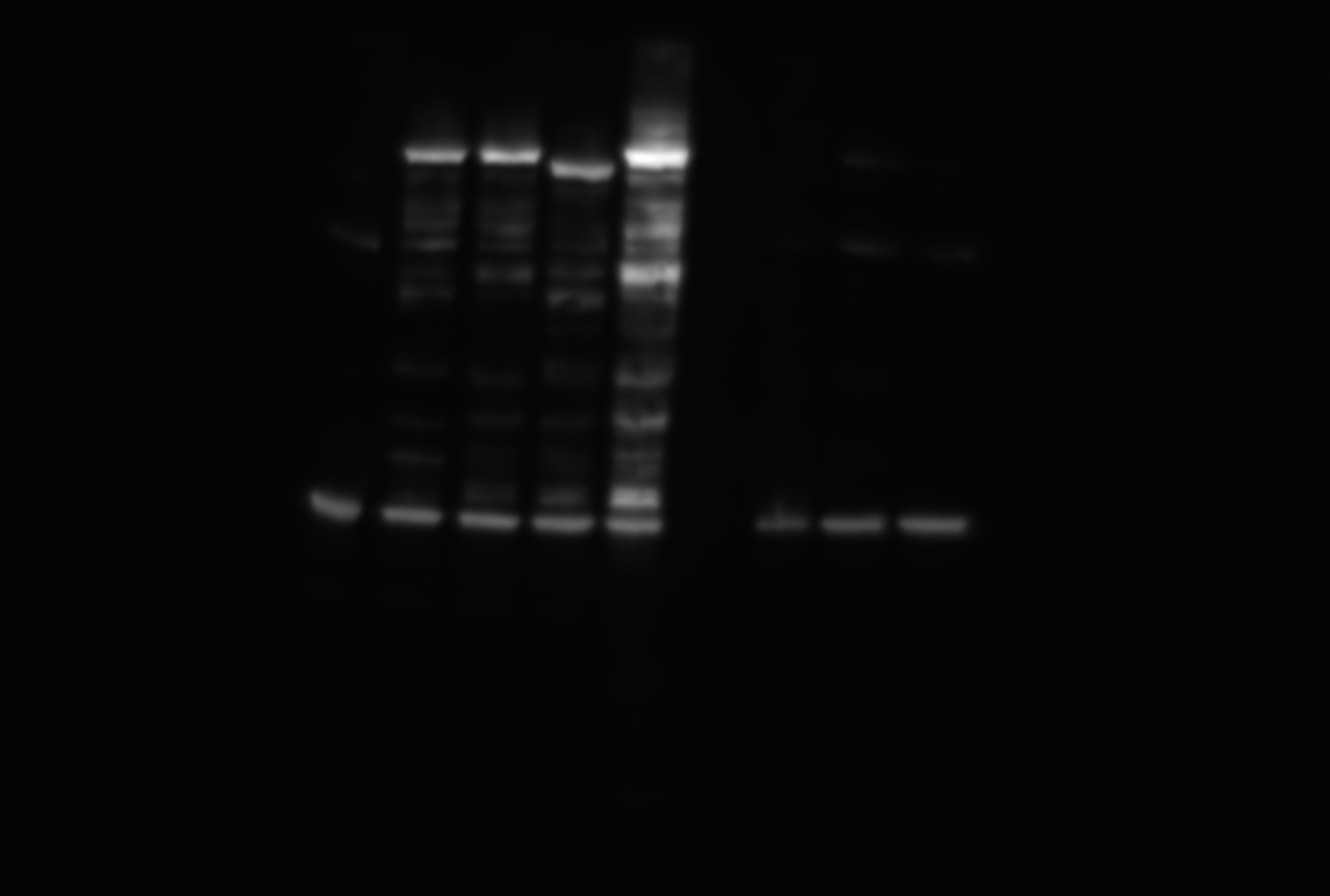

Supplement: Supplementary file 1 [file LSA-2022-01686_SdataF1_FS2_FS3.zip › Raw blots and source data Kopie/Fig S2/Eplin OE KO/Eplin OE KO/GAPDH zu EPLIN001-01.tif]

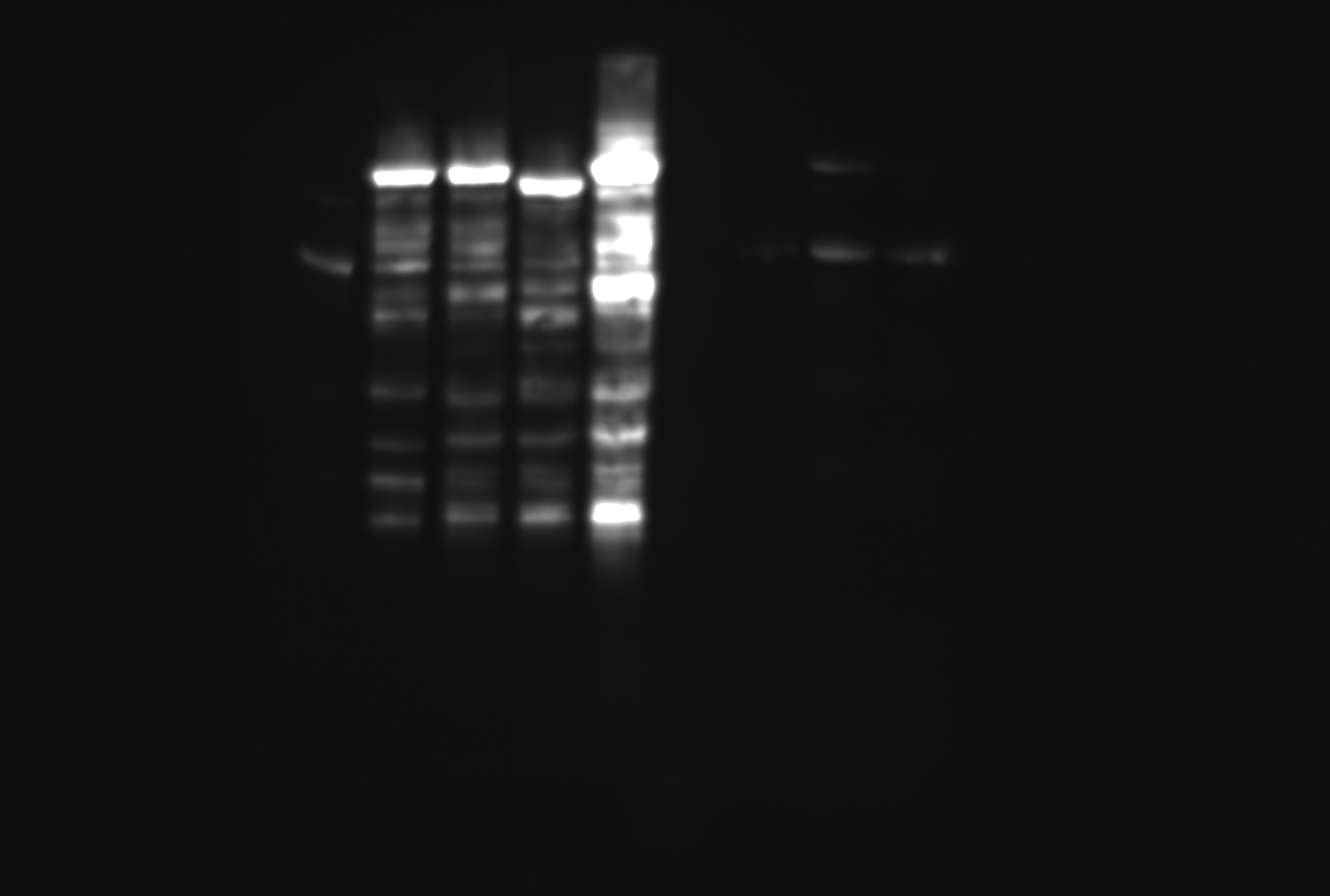

Supplement: Supplementary file 1 [file LSA-2022-01686_SdataF1_FS2_FS3.zip › Raw blots and source data Kopie/Fig S2/Eplin OE KO/Eplin OE KO/eplin001-03.tif]

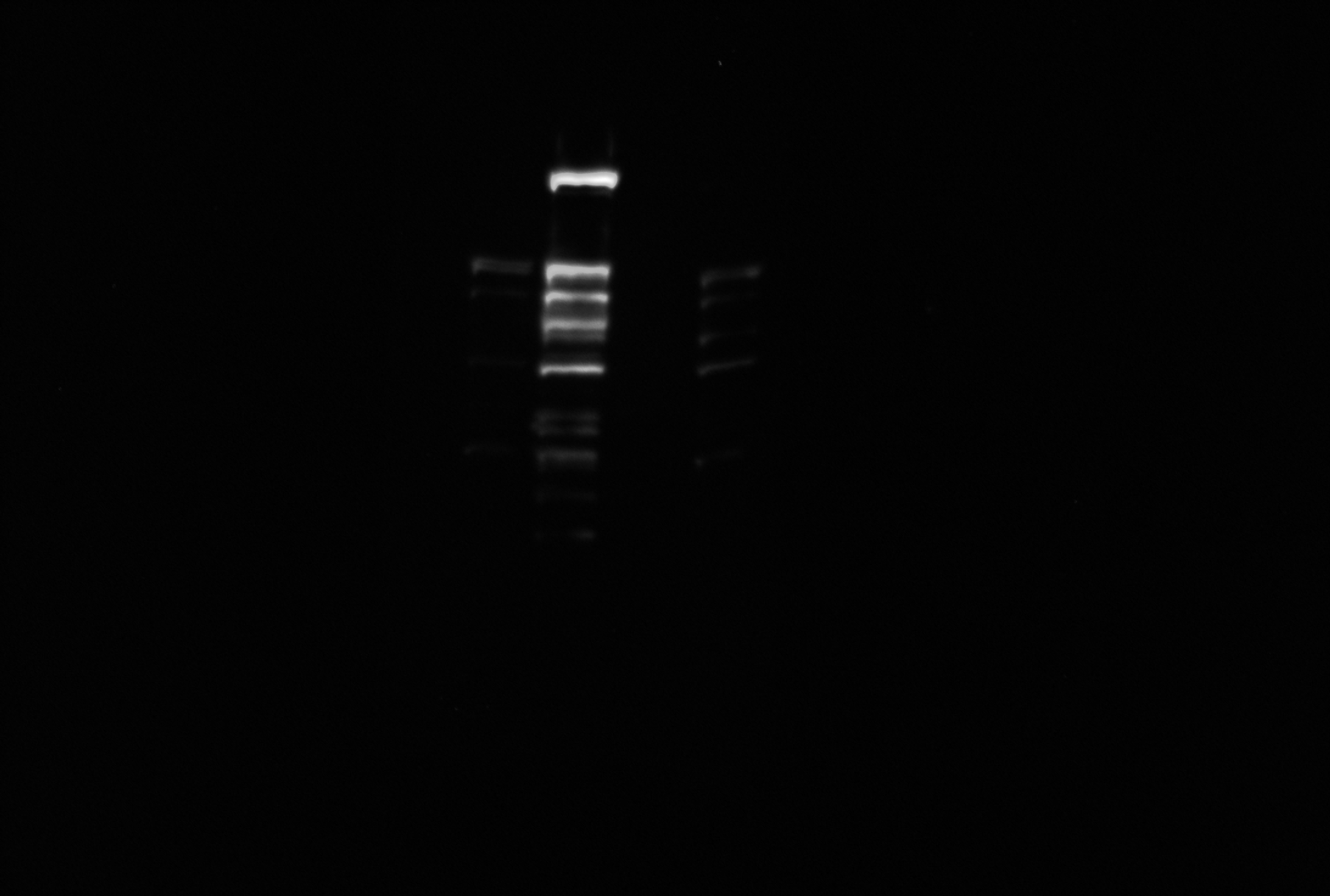

Supplement: Supplementary file 1 [file LSA-2022-01686_SdataF1_FS2_FS3.zip › Raw blots and source data Kopie/Fig S2/S9/S9/stable/sept9 v2001-01.tif]
