# Supplementary material for: The concerted action of SEPT9 and EPLIN modulates the adhesion and migration of human fibroblasts
Source: Life Sci Alliance. 2024 May 7;7(7):e202201686. doi: 10.26508/lsa.202201686 (PMC11077590; doi:10.26508/lsa.202201686)
Supplement: Supplementary file 6 [file LSA-2022-01686_TableS1.docx]

**Supplementary Information**

**Suppl. Table 1:** Oligonucleotides as primers for PCRs (in 5’-3 orientation)

| Name | Sequence (5’-3’) | Construct/Purpose |
| --- | --- | --- |
| GSTEPLa_SbfI_fw | gatcCCTGCAGGctGAAAATTGTCTAGGAGAATCCAGGC | GST-EPLIN |
| GSTEPLa_AscI_rv | cgatGGCGCGCCTCACTCTTCATCCTCATCCT | GST-EPLIN |
| EpLIM_BamHI_fw | gtcaGGATCCgagacctgcgtggaatgtcag | GST-EPLIN_LIM |
| EplLIM_AscI_rv | gtaGGCGCGCCTCAagatttaaagagttgattgaagtga  ggc | GST-EPLIN_LIM |
| Sept9-Sfi1for | CCGAAGGCCAGCACGGCCGAAAACCTGTACTTCCAGGGT AAGAAGTCTTACTCAGGAGG | GST-SEPT9,  His-SEPT9 |
| Sept9-Sfi1rev | CTGTGGGCCAAAAAGGCCTTATCACATCTCTGGGGCTTCTGG | GST-SEPT9,  His-SEPT9 |
| His-EPLIN_Sfi_fw | gcatcGGCCAGCACGGCCGAAAATTGTCTAGGAGAATC  CAGGC | His-EPLIN |
| His-EPLIN_Sfi_rv | gtactGGCCAAAAAGGCCTCACTCTTCATCCTCATCCTC  ATCA | His-EPLIN |
| GFP-EPLIN_SalI_fw | ctagGTCGACATGGTGAGCAAGGGCGAGG | EGFP-EPLIN |
| GFP-EPLIN_NheI_rv | ctagGCTAGCtcactcttcatcctcatcctcatcataata  c | EGFP-EPLIN |
| iRFP_Crispr.rev | GACTGAATTCTTAGCGTTGGTGGTGGGC | pSpCas9(BB)-2A-iRFP |
| T2A_iRFP-fw | GACTGAATTCGGCAGTGGAGAGGGCAG | pSpCas9(BB)-2A-iRFP |
| SEPT9_X4seq.fw | GTTCCGCTCTAACTCCTCTGC | sequencing of SEPT9 Exon4 |
| SEPT9_X4seq.rv | GCTGGTTTCCGGCATTGG | sequencing of SEPT9 Exon4 |
| SEPT9_X6seq.fw | GTGCAGATATTGAGGAGAAAGGCG | sequencing of SEPT9 Exon6 |
| SEPT9_X6seq.rv | GGCCTCACCAGTTCTCGTTG | sequencing of SEPT9 Exon6 |
| S9_Gdom_sfi_fw | GCATCGGCCAGCACGGCCCAGGGCTTCGAGTTCAACAT  CATG | His-SEPT9 ΔNΔC |
| S9_Gdom_sfi_rv | CATGAGGCCAAAAAGGCCTCACTCGTTGAGGCGCTTCA  CAC | His-SEPT9 ΔNΔC |
| S9_Sfi_fw | CCATGAGCAGCCATCATCATC | His-SEPT9 ΔC |
| S9_sfi_rv | GTTAGCAGCCGGATCCGTTG | His-SEPT9 ΔN |
| Primer 1_EPLIN_splic1.fw | CATGGACGAGCTGTACAAGTCC | EGFP-EPLIN ΔLIM |
| Primer 2_EPLIN_splic2.rv | CATCATAGTTGCCCTTTCTTGCAGGTGCCTGAAAC | EGFP-EPLIN ΔLIM |
| Primer 3_EPLIN_splic3.fw | CAGGCACCTGCAAGAAAGGGCAACTATGATGAAGGC | EGFP-EPLIN ΔLIM |
| Primer 4_EPLIN_splic4.rv | CTAGATCCGGTGGATCCCGG | EGFP-EPLIN ΔLIM |
| GFP-LIM_BspEI_fw | gtcaTCCGGAgagacctgcgtggaatgtcagaag | EGFP-EPLIN_LIM |
| GFP-LIM_SalI_rv | catgGTCGACaTCAagatttaaagagttgattgaagtga  ggc | EGFP-EPLIN_LIM |
