# Supplementary material for: The concerted action of SEPT9 and EPLIN modulates the adhesion and migration of human fibroblasts
Source: Life Sci Alliance. 2024 May 7;7(7):e202201686. doi: 10.26508/lsa.202201686 (PMC11077590; doi:10.26508/lsa.202201686)
Supplement: Supplementary file 7 [file LSA-2022-01686_TableS2.docx]

**Suppl. Table 2: Primary and secondary antibodies (Abs) used for immunofluorescence (IF) or Western Blotting (WB)**

| Product | Host | Dilution  IF | Dilution  WB | Supplier | Product ID |
| --- | --- | --- | --- | --- | --- |
| Primary Abs |  |  |  |  |  |
| α-SEPT9  (N-term) | rabbit | 1:100 | 1:5000 | Prof. Krauss, Leibniz Institute of Molecular Pharmacology, Berlin | |
| α-SEPT9 | mouse | - | 1:3000 | Sigma Aldrich (2C6) | # WH001-  0801M1 |
| α-SEPT9  (G-domain) | rabbit | 1:100 | 1:5000 | Bethyl Laboratories | # A302-354A |
| α-SEPT7 | rabbit | 1:100 | 1:5000 | Sigma-Aldrich | # HPA029524 |
| α-β-Actin | mouse | 1:100 | 1:2000 | SCBT (E-10) | # sc-365791 |
| α-EPLIN | rabbit | 1:200-1:100 | - | FineTest | # FNab02812 |
| α-EPLIN | mouse | 1:100 | 1:2500 | SCBT (20) | # sc-136399 |
| α-Paxillin | mouse | 1:100 | 1:3000 | SCBT (D-9) | # sc-365174 |
| α-VASP | rabbit | 1:100 | - | Thermo Fisher Scientific | #MA5-35728 |
| α-GAPDH-HRP | mouse | 1:100 | 1:2500 | SCBT (0411) | # sc-47724 |
| α-His_6_ | mouse | - | 1:5000 | Sigma Aldrich (HIS-1) | # H1029 |
| Secondary Abs |  |  |  |  |  |
| α-mouse-Alexa Fluor^®^ 488 | goat | 1:1000-1:500 | - | Thermo Fisher Scientific | # A28175 |
| α-mouse-Alexa Fluo^®^ 555 | goat | 1:1000-1:500 | - | Thermo Fisher Scientific | # A32727 |
| α-mouse-Alexa Fluor^®^ 647 | goat | 1:1000-1:500 | - | Thermo Fisher Scientific | # A32728 |
| α-rabbit-Alexa Fluor^®^ 488 | goat | 1:1000-1:500 | - | Thermo Fisher Scientific | # A27034 |
| α-rabbit-Alexa Fluor^®^ 555 | goat | 1:1000-1:500 | - | Thermo Fisher Scientific | # A27039 |
| α-mouse-HRP | goat | - | 1:5000 | Sigma Aldrich | # A4416 |
| α-rabbit-HRP | goat | - | 1:5000 | Bio-Rad | # 170-6515 |
