# Supplementary material for: The concerted action of SEPT9 and EPLIN modulates the adhesion and migration of human fibroblasts
Source: Life Sci Alliance. 2024 May 7;7(7):e202201686. doi: 10.26508/lsa.202201686 (PMC11077590; doi:10.26508/lsa.202201686)
Supplement: Supplementary file 8 [file LSA-2022-01686_TableS3.docx]

**Suppl. Table 3: Sequences of sgRNAs for CRISPR/Cas9 gene knockout** (capital letters

mark the targeting sgRNA sequence, whereas lowercase letters indicate overhangs for cloning into pSpCas9(BB)2A via BbsI.

| Name | Sequence (5’-3’) | PAM site | On target value [%] | Off-target value [%] |
| --- | --- | --- | --- | --- |
| SEPT9_sgRNA_Exon 4B_fw | caccgGCCTGCATCACGGAACGAGA | AGG | 62.5 | 90.3 |
| SEPT9_sgRNA_Exon 4B_rv | aaacTCTCGTTCCGTGATGCAGGCc |  |  |  |
| SEPT9_sgRNA_Exon 6E_fw | caccgTGACAGTGATTGACACACCA | CGG | 76.7 | 66.4 |
| SEPT9_sgRNA_Exon 6E_rv | aaacTGGTGTGTCAATCACTGTCAc |  |  |  |
